# Selenium, TGF-Beta and Infectious Endemic Cardiopathy: Lessons from Benchwork to Clinical Application in Chagas Disease

**DOI:** 10.3390/biom12030349

**Published:** 2022-02-23

**Authors:** Tania C. Araujo-Jorge, Maria Teresa Rivera, Jean Vanderpas, Luciana R. Garzoni, Anna Cristina C. Carvalho, Mariana C. Waghabi, Marcelo T. Holanda, Mauro F. F. Mediano, Alejandro M. Hasslocher-Moreno, Maria da Gloria Bonecini-Almeida, Roberto M. Saraiva, Roberto R. Ferreira

**Affiliations:** 1 Laboratory of Innovations in Therapies, Education and Bioproducts, Oswaldo Cruz Institute (LITEB-IOC/Fiocruz), Oswaldo Cruz Foundation (Fiocruz), Avenida Brasil 4365, Manguinhos, Pavilhão Cardoso Fontes, Sala 64, Rio de Janeiro 21040-360, Brazil; largarz@ioc.fiocruz.br (L.R.G.); anna.carvalho@ioc.fiocruz.br (A.C.C.C.); robertoferreira.ioc@gmail.com (R.R.F.); 2 École de Santé Publique, Campus Erasme, Université Libre de Bruxelles, 808 Route de Lennik, 1070 Bruxelles, Belgium; mariateresa.rivera@skynet.be (M.T.R.); jean.vanderpas@skynet.be (J.V.); 3 Laboratory of Functional Genomic and Bioinformatics, Oswaldo Cruz Institute (LGFB-IOC/Fiocruz), Oswaldo Cruz Foundation (Fiocruz), Avenida Brasil 4365, Manguinhos, Pavilhão Leonidas Deane, Rio de Janeiro 21040-360, Brazil; mariana@ioc.fiocruz.br; 4 Laboratory of Clinical Research in Chagas Disease, Evandro Chagas National Institute of Infectious Diseases (INI), Oswaldo Cruz Foundation (Fiocruz), Avenida Brasil 4365, Manguinhos, Rio de Janeiro 21040-360, Brazil; marcelo.holanda@ini.fiocruz.br (M.T.H.); mauro.mediano@ini.fiocruz.br (M.F.F.M.); alejandro.hasslocher@ini.fiocruz.br (A.M.H.-M.); roberto.saraiva@ini.fiocruz.br (R.M.S.); 5 Laboratory of Immunology and Immunogenetics, Evandro Chagas National Institute of Infectious Diseases, Oswaldo Cruz Foundation, Avenida Brasil 4365, Manguinhos, Rio de Janeiro 21040-360, Brazil; gloria.bonecini@ini.fiocruz.br

**Keywords:** myocardiopathy, infection, pathogenesis, selenoproteins, TGFbeta signaling, translational research

## Abstract

For over 60 years, selenium (Se) has been known as an essential microelement to many biological functions, including cardiovascular homeostasis. This review presents a compilation of studies conducted in the past 20 years related to chronic Chagas disease cardiomyopathy (CCC), caused by *Trypanosoma cruzi* infection, a neglected disease that represents a global burden, especially in Latin America. Experimental and clinical data indicate that Se may be used as a complementary therapy to prevent heart failure and improve heart function. Starting from the main questions “*Is Se deficiency related to heart inflammation and arrhythmogenesis in CCC*?” and “*Could Se be recommended as a therapeutic strategy for CCC*?”, we show evidence implicating the complex and multidetermined CCC physiopathology, discussing its possible interplays with the multifunctional cytokine TGF-β as regulators of immune response and fibrosis. We present two new proposals to face this global public health challenge in vulnerable populations affected by this parasitic disease: fibrosis modulation mediated by TGF-β pathways and the possible use of selenoproteins as antioxidants regulating the increased reactive oxygen stress present in CCC inflammatory environments. We assess the opportunity to consider the beneficial effects of Se in preventing heart failure as a concept to be applied for CCC patients.

## 1. Epidemiology and Relevance of Chagas Disease and Chronic Chagas Cardiopathy: A Neglected Tropical Disease

Acute or chronic inflammation of the heart are important causes of pathological outcomes implicating heart function and failure and cardiovascular diseases (CVD) [1]. Endocarditis, myocarditis, epicarditis and/or pericarditis occur as consequences of infectious or non-infectious heart damage [1,2]. Infectious cardiopathies occur when viruses, bacteria, protozoa, or fungi interact and enter one or more heart structures, triggering local and/or systemic inflammation. From the infections in the three main heart layers, myocarditis is more frequently caused by cardiotropic viruses (historically, Coxsackieviruses and adenoviruses have been commonly suspected in myocarditis patients in North America and Europe and, less frequently, parvovirus and human herpes viruses [3]), bacteria (e.g., *Corynebacterium diphtheriae*, *Staphylococcus aureus*, *Borrelia burgdorferi*, *and Ehrlichia species* [4,5]) and by specific endemic parasites such as *Trypanosoma cruzi (T. cruzi)* and *Babesia microti*, as well as fungal infection by *Cryptococcus* [6]. 

Human chronic Chagas disease (CD) cardiomyopathy (CCC) is caused by *T. cruzi* and is the most relevant infectious heart condition in Latin America, due to CD distribution over 21 countries affecting 6–7 million people worldwide and leading to 12,000 deaths/year [7,8]. CD is classified as a neglected tropical disease (NTD) [7,8], a concept that links the pharmaceutical industry’s disinterest in poverty-centered target populations. CD is now a global burden due to migration from Latin America to other continents, including Europe, North America, and Asia [9]. Trypanocide treatment with benznidazole or nifurtimox is recommended to prevent or reduce disease progression in both the acute and chronic phases [8]. 

CD is neglected in multiple dimensions. The first dimension of CD neglect is related to the volume of basic and translational research conducted worldwide to produce new knowledge concerning the physiopathology mechanisms and possible treatments for CD (Table 1). Compared to basic and clinical research on cancer, for example, CD received less than 0.5% of the scientific attention, even less than tuberculosis (6%), HIV (3.4%) or malaria (2.2%). Recently, the huge worldwide effort to understand COVID-19 issues and to search for vaccines and drug treatments led to 193,338 publications in PubMed (4.3%) in just 2 years of studies. Assays in people and patients dealing with observational, retrospective, prospective or randomized clinical trials (RCT) are even more scarce for CD, thus accounting for less than 0.1% of the studies in cancer (Table 1). A significant difference in investment in CD compared to malaria or HIV should be highlighted.

## 2. Selenium Roles in Myocardiopathies via Selenoproteins: The Relevance of Adequate Selenium Levels 

Selenium (Se) is a nutritional essential trace element relevant to some myocardiopathies [10,11], a condition that was first depicted in studies concerning Keshan cardiomyopathy. This cardiopathy is related to endemic Se nutritional deficiency in Chinese regions where the soil is especially poor in Se, and consequently vegetable foods and animal nutrition are insufficient in Se [11]. Observational evidence on the benefit of sodium selenite in Keshan’s cardiomyopathy dates to 1979. Since then, the association of selenoenzymes with cardiovascular physiology has been ratified (see Supplementary Table S1 summarizing selected references on this subject). Clinical trials published in Sweden between 2013 and 2018 confirmed the efficacy of a 4-year supplementation with Se and coenzyme Q10 (coQ10) in reducing cardiovascular mortality in the elderly, followed up for 12 years after the intervention by additional changes in important biomarkers of inflammation and of cardiac injury [12]. Recent papers [12,13], reviews and meta-analyses [14,15,16,17] implicate Se supplementation as a therapeutic strategy for preventing heart disease (Table S1). Evidence suggests a role for individual selenoproteins in cardiovascular diseases, among the 25 selenoproteins present in humans and 24 in mice [18]. Since chronic myocarditis depends on sustained chronic inflammation, and the immune system is affected both by dietary Se levels as well as by selenoprotein expression [18], the search for relationships between Se and myocardiopathies is a promising route. 

The role of Se in CD is also a neglected subject, as indicated by basic and applied studies (Table 1); when all the 38,567 Se studies carried out since 1909 are compared to the amount of research on CD and Se, records found in PubMed account for 0.06%. Translation of science from bench to public health policies is even lower (0.03%) when comparing records retrieved from the Clinical Trials platform. We were proud to deliver the first RCT for CD and Se, which will be further discussed in this review [19]. 

## 3. Chagas Disease Physiopathology: Complex and Multi-Determinate with Emerging Roles for Selenium and TGF-Beta

CCC is a complex disease with multiple determinants and its physiopathology was extensively reviewed [20,21]. After 1–3 months and a mostly benign acute phase in humans, CD follows a silent and asymptomatic indeterminate phase in immunocompetent hosts and can remain in that stage for the life span of the affected person. However, due to triggers still unknown (s), a chronic and slow progression may happen in about 25–30% of the seropositive cases, evolving from a simple chronic infection to an important organic disease in which two major clinical forms may occur: a digestive and/or a cardiac form [20,21]. 

**Figure 1:**
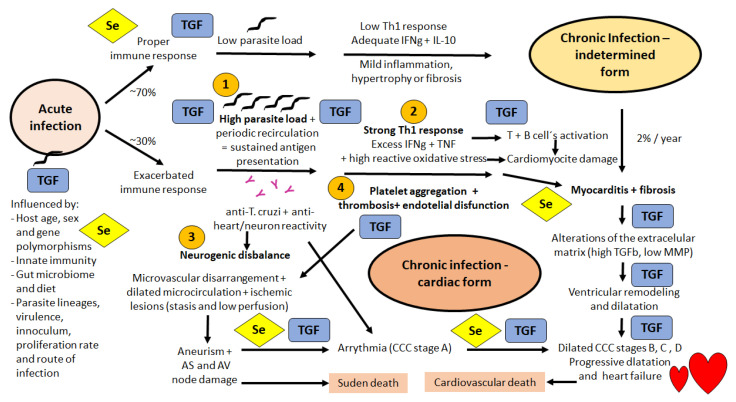
Schematic view of the Chagas disease Chronic Cardiopathy (CCC) physiopathology mechanism showing all the possible implications of selenoproteins involvement (marked Se in a yellow diamond) and/or TGF-beta related effects (marked TGF in a blue rectangle) considering experimental and clinical data available in the literature. In immunocompetent mammals, the acquired adaptive and specific immune response commonly develops at a proper level following an acute phase that lasts about 1 to 3 months in humans and triggers a strong innate immune response. Such mechanisms maintain a low parasite load and an adequate IFN-gamma and IL-10 response (low Th1 type) in about 70% of the cases, sustaining seropositive individuals in an indeterminate form in the chronic phase, with a barely detected parasitemia. However, in about 30% of the human cases, an abnormal cardiac form develops in the chronic phase involving at least four main pathogenic mechanisms (marked 1,2,3, and 4 in the figure): (**1**) direct heart cell damage caused by parasite infection as nests in the myocardium; (**2**) direct damage caused by focal CD8-driven myocytolysis and muscle cell tissue substitution by fibrosis; (**3**) a neurogenic disbalance caused by excessive parasympathetic stimulation by neurotransmitter agonist action of muscarinic-like antibodies produced by *T. cruzi*-infected persons; and (**4**) endothelial dysfunction with microvascular compromise. The major clinical outcomes are sudden or cardiovascular death, stroke, CCC progression and typical abnormalities in electrocardiograms presented as bradyarrhythmias, tachyarrhythmias, or even complex arrhythmias. The important factors affecting the balance between indeterminate or CCC progression forms are: (i) inflammation x parasite load and (ii) maintenance and repair for homeostasis x stressed or malfunctioning tissue sustaining chronic inflammation. The authors expanded and produced Figure 1.

For our goal of focusing on CCC, it is worth mentioning the four main concomitant mechanisms undergoing CCC pathogenesis (Figure 1): (1) a direct heart cell damage caused by parasite infection as nests that slowly but not synchronically disrupts the myocardium; (2) direct damage caused by focal CD8-driven infiltration with myocytolysis and muscle cell tissue substitution by focal fibrosis; (3) a neurogenic disbalance caused by excessive parasympathetic stimulation through neurotransmitter agonist action of muscarinic-like antibodies produced by *T. cruzi*-infected persons; and (4) endothelial disfunction with microvascular compromise, microthrombi and focal ischemia. A recent review of CD immunology summarizes the relevance of the immune system both to control the infection and drive it into a benign chronic indeterminate phase, or to foster and sustain chronic inflammation that leads to CCC pathogenesis [22]. A successful control of the acute phase is engendered by macrophages producing IL-2, NO, CXCL9 and CXCL10, lymphocytes producing TNF, IFN-γ and IL-17, and dendritic cells producing IL-12 and TNF. The indeterminate form of the chronic phase is characterized by the low levels of IFN-γ and TNF, and adequate levels of IL-10 and IL-17, while the cardiac form of the chronic phase presents high levels of IFN-γ, TNF, IL-1β and TGF-β, and low IL-18 levels [22]. Figure 1 shows the steps where TGF-β is possibly involved, some of them also possibly influenced by one or more selenoproteins, and then depending on adequate Se intake and bioavailability. Despite Se being a constitutive element of the selenocysteine in amino acids, selenoproteins ultimately are sensitive to Se deficiency at <64 mcg/day. 

## 4. Selenium, TGF Beta and Chagas Cardiomyopathy: First Data, Animal Models and Basic Science Research

Our studies on Se and CD [23,24,25] were conducted in parallel to our research on TGF-β [26,27,28,29,30,31] and only recently, we have started to unveil the possible connections between events and mechanisms common to elements from the two fields of interest. Table 2 shows some of these mechanisms and lists the respective references. 

Most of these parameters are simultaneously influenced by the supplementation/deficiency in Se and the modulation of TGF-β−signaling, as summarized in Table 2. However, the possibility of starting clinical trials to test the effect of Se supplementation in CD patients, and the present unavailability of safe TGF-β modulators for studies in humans, drove our focus to Se effects in CD patients [19] and to further analysis of TGF-β modulation in pre-clinical experimental models [27,28,29,30,31]. At this point, the studies were developed according to the following different strategies: (a) basic research concerning TGF-β signaling effects in fibrosis and arrhythmia that generated interesting results [27,28,29,30,31]; (b) efforts to make translational proof-of-concept research on the presumed benefit of Se supplementation in CD patients. 

## 5. Selenium and Chagas Cardiomyopathy: From Observational Studies to Basic Research and Clinical Trials 

The first concept that Se could be involved in CCC occurred in 1998 following the studies on Kashin-Beck osteoarthropathy (another endemic Se-deficiency disease) performed within the scope of the collaboration between Belgian and Chinese colleagues [38]. These Tibetan studies demonstrated that the addition of Se to the diet provides a correction of biochemical markers of selenium deficiency, even if one year of Se versus placebo supplementation in a randomized clinical trial did not show a therapeutic benefit on osteoarthropathy of children once combined iodine deficiency had been corrected [39]. A question drove our first work: *Could severe CCC be associated with Se deficiency?* We then (i) compared circulating levels of Se in different stages of CCC [23], showing that lower Se levels were more frequent in patients with more severe CCC, (ii) started experimental studies in mice fed with Se-deficient chow, infected in vivo [24], and (iii) studied myocardiopathy in the chronic phase after supplementing the diet with Se [25]. Table 2 shows the different events studied so far. 

Some selenoproteins are implied in cytokine expression and modulation, as shown in recent metallomics studies [40]. The authors studied pigs with dietary Se deficiency or supplementation and found that Se deficiency decreased significantly the Se concentration and selenoprotein expression in the spleen, blocked the glutathione and thioredoxin antioxidant systems, and led to redox imbalance. Se deficiency also increased inflammation by activating the NF-κB and HIF-1α transcription factors, thus increasing pro-inflammatory cytokines (IL-1β, IL-6, IL-8, IL-17, and TNF-α) and decreasing anti-inflammatory cytokines (IL-10, IL-13, and TGF-β). Studies in chickens [41] showed that Se deficiency influenced the expressions of 24 selenoproteins and 10 cytokines (including IL-2, IL-4, IL-8, IL-10, IL-12β, TGF-β4, and IFN-γ) in erythrocytes, revealing a relationship between Se and the immune system. These results reinforce the importance of studying immune and antioxidative biomarkers in CD patients during the natural course of CCC development, as well as under the effect of Se treatment [19]. 

A first hypothesis is that TGF-β levels could also correlate to some selenoprotein levels and Se status. We showed that CD patients displayed higher TGF-β levels [26] and lower Se levels [23] in the severe CCC stages, as compared to the asymptomatic indeterminate form. These data were different from the deficiency and supplementation effects in the pig studies [41]. Based on results showing that Se deficiency activated the NF-κB and HIF-1α transcription factors, these authors concluded that Se deficiency induces spleen injury by regulating seleno-proteins, oxidative stress, inflammation, and apoptosis. In addition, Se deficiency led to an increase in pro-inflammatory cytokines (IL-1β, IL-6, IL-8, IL-17, and TNF-α), a decrease in anti-inflammatory cytokines (IL-10, IL-13, and TGF-β) and increased expression of the downstream genes COX-2 and iNOS (*p* < 0.05), induced inflammation. Se deficiency also induced apoptosis through the mitochondrial pathway, upregulated apoptotic genes (Caspase3, Caspase8, and Bak), and downregulated significantly antiapoptotic genes (Bcl-2) at the mRNA level. Then, the relationship between Se and TGF-β could be part of a homeostatic response, given that TGF-β1 is able to modulate the expression of selenoprotein P [42], the main plasma selenoprotein that drives Se to the tissues. In severely compromised cardiac CD persons, TGF-β is activated, as shown by phospho Smad 2 staining in heart biopsies [26], which could lead to increased selenoprotein P expression and drive more Se to cardiac tissue to increase the local ability to face the oxidative stress that occurs in the inflamed myocardium. Another intriguing result was obtained in a study showing that TGFβ levels decreased in stages C and D [43], a difference that was interpreted as being due to the different age range of the patients enrolled in the study and in the different treatment regimen with carvedilol or spironolactone that were clinically introduced in the following decade. 

In fact, any direct interpretation of Se and TGF-β in CD physiopathology must be done with caution, since both Se and TGF-β are known to present bi-modal U-shaped effects, depending on their concentration in microenvironments [44]. However, this review argues precisely for the need for more precise observations on this subject. Other interplays between selenoproteins and TGFβ- mediated effects were shown for thyroid fibrosis [45] and tumors studied experimentally [46]. As pointed out by many authors, “the impact of Se administration should be considered in relation to its apparent U-shaped effects, i.e., exhibiting major advantages in Se-deficient individuals but specific health risks in those with Se excess” [44]. 

## 6. Proof of Concept Studies in Humans: Chagas Disease Progression Implicates Selenium/Selenoproteins and TGF-β Signaling

As stated above, our first studies in CD patients showed lower Se levels [23] and higher TGF-β levels [26] in the most severe CCC stages, as compared to the asymptomatic indeterminate form. By that time (1998–2002), the new classification of CCC stages that are now used in Brazil was not yet developed [47,48,49]. In Table 3, we revisited those data according to the new classification that drove our choices for the RCT designed in 2005–2009 [50] and effectively performed in 2014–2020 [19]. 

So far, despite the differences that we observed in Se levels in heathy and CD patients in two Brazilian regions (72 ± 10 ng/mL in Rio de Janeiro city and 55 ± 10 ng/mL in Belo Horizonte city), our studies have shown that there is no systematic endemic Se deficiency in Brazilian areas where CCC is prevalent. The biochemical measurements of serum Se levels in CD patients in the indeterminate form and in the early cardiac form at stages A and B1 were within the ordinary range [19,23], contrasting with the reduction in patients with severe cardiac dysfunction CCC [23]. A meta-regression analysis in a recent systematic review [15] revealed a statistically significant linear dose-response relationship between blood selenium concentration and CVD incidence, but not CVD mortality. The risk of CVD incidence was reduced by 15% (RR¼0.85, 95% CI: 0.76–0.94) per 10 mcg increment in blood selenium concentration. This study considered low Se intake (<50 mcg/L) or low Se status (serum level < 100 mcg/L) as a risk factor in Asian and European populations, where protective effects of selenium were found [15]. 

## 7. Lessons from the First and Unique Se Trial in Chagas Disease

As shown in Table 2, preclinical studies showed the benefit of Se treatment in preventing and reversing cardiac and digestive injuries [24,25,34]. We also found a correlation between Se levels and the left ventricular ejection fraction (LVEF) value [23]. Since LVEF is the best indicator of progression of heart dysfunction [47,48,49] and improves after micronutrient supplementation in patients with non-infective HF [51], we chose this endpoint as a major outcome for an STCC (Selenium Treatment and Chagasic Cardiopathy) trial [19]. Considering there is no systematic study of Se levels in soil and in diets in different geographic regions where CD is endemic, it is not known if a combined selenium deficiency occurs or not in Brazilian areas known to be endemic for Chagas cardiomyopathy. The differences observed in Se levels in healthy and CD patients in the cities of Rio de Janeiro and Belo Horizonte [23] indicates that this possibility would deserve further clarifying studies. In 2004, we designed a RCT as a proof of concept on the efficacy of Se in the progression of mild or moderate cardiac CD [50]. The STCC trial was approved by the Brazilian Ethical Committee in 2005, registered both at the Clinical Trials and the Brazilian Regulatory Agency (ANVISA) in 2009, and started after the production of the prepared batch with good manufacturing practices (GMP) by Catalent, the industrial partner in 2013 [50]. STCC was a single-center, prospective, double-blinded, placebo-controlled, phase III, superiority randomized clinical trial that aimed to estimate the effect of Se treatment on prevention of cardiac disease progression in patients with the CD cardiac form [50].

**Figure 2:**
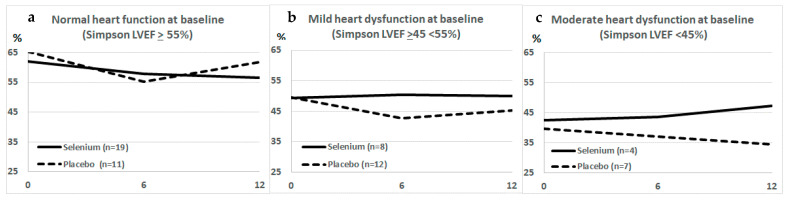
Changes in left ventricular ejection fraction (LVEF) after 6 and 12 months in Chagas disease patients participating in STCC, after Se (solid lines) or Placebo (traced lines). The effect of Se was statistically significant only in panel (**c**), in patients starting the trial with a moderate heart disfunction and LVEF lower than 45%. Patients with normal heart function (LVEF ≥ 55%, panel (**a**) or with mild heart disfunction (LVEF between 45 and 55%, panel (**b**) did not show a significant effect after Se treatment.

Completed in 2018 and recently published [19], STCC showed the safety of Se administration in patients. No significant difference in mean LVEF between the two groups at the end point of one year was shown, but in the CCC stage B2 subgroup (LVEF < 45%) Se administration resulted in statistically significant longitudinal changes in mean LVEF (Figure 2). As shown in the STCC results [19], the study of individual LVEF trajectories showed that only in the Se group were there ascending curves [19] that rose more than 10 percentage points in a one-year follow-up [19]. 

The main conclusions of STCC were that, in the subgroup of patients at CCC stage B2, a potential beneficial effect of Se was observed and that complementary studies were necessary to explore diverse Se doses and/or associations in different CCC stages (B2 and C), as well as in A and B1 stages with longer follow-up. These data justify continuing the research along this line by adding future RCTs that (a) double the Se dose and add coQ10, as reported [12]; treat patients (b) for longer periods of time—2 years and (c) in more than one clinical center; and (d) include patients pretreated with BZ without seroconversion (seropositive in two tests for CD). It would be important to expand the study geographically (other Brazilian states, Bolivia, and Spain) and in clinical cardiac subgroups (A, B1, B2 and C). If validated, Se treatment can be made available for primary care in health systems in the line of care for chronic CD patients [49].

A direct correlation between serum Se levels and progression of heart disfunction, especially heart failure, is still under debate [14,15,16,17,52,53,54]. STCC also showed scattered basal levels of Se ranged from 40 to 150 ng/mL, a variability that deserves further studies for a clearest picture related to selenoprotein genetic polymorphisms and/or regional diets varying in Se content. The table in the supplementary material summarizes the main references that support this discussion. Serum Se concentration (deficiency) was <70 mcg/L in 20.4% of the patients studied by Nils Bomer et al. [52], “who were older, more often women, had worse New York Heart Association class, more severe signs and symptoms of heart failure and poorer exercise capacity (6-min walking test) and quality of life (Kansas City Cardiomyopathy Questionnaire). Se deficiency was associated with higher rates of the primary endpoint, a composite of all-cause mortality and hospitalization for heart failure, and with all-cause mortality” [52]. A study by Al-Mubarak et al. [54] in the same group, focused on heart failure, one of the main outcomes in CCC. Authors addressed that “suboptimal selenium levels (<100 mcg/L) are prevalent in more than 70% of patients with heart failure and were associated with lower exercise capacity, lower quality of life, and worse prognosis.” In the STCC study [19], 61% of CD patients showed Se levels < 100 mcg/L, clearly indicating that Se was suboptimal in most of the participants, but only 7% were <70 mcg/L. Fifteen years ago, in our first Se study with CD patients [23] 96% of the participants showed Se levels < 100 mcg/L and 52% < 70 mcg/L. In a Chinese populational study including 411 patients with heart failure [53], the mean serum level of Se was of 68.3 ± 27.7 mcg/L. This study showed that lower serum Se level was significantly associated with increased risk of all-cause mortality in patients with heart failure, comparing patients in the highest quartile of Se levels (94.15–116.7 ng/mL), to those with lowest quartile (17.40–44.35 ng/mL). 

A recent meta-analysis [15] reviewed 13 out of 1811 observational studies and RCTs to evaluate the association between Se status and incidence and mortality of CVD. The authors concluded that “physiologically high Se levels in blood and toenail is associated with decreased CVD incidence and mortality; however, the daily Se intake should be within the recommended daily allowance (50–300 mcg/day) to prevent the harmful effects of Se that may occur at levels beyond 300 mcg/day.” This review engaged Chinese, Swedish, African, and Russian co-authors that asserted the “need for further studies on specific types of CVD and Se as some seem to be more associated with Se than others.” They found a reduced risk of CVD incidence and mortality in subjects with physiologically high Se status compared to low Se status. The review included studies that used at least two Se determinations and doses, besides the already mentioned 15% decreased risk of CVD incidence per 10 mcg increment in blood Se concentration; in addition, a significant nonlinear dose-response relationship was found between CVD mortality and increased blood Se concentration with the lowest risk at the 30–35 mg increment in blood Se. 

Considering the geographical differences in serum Se observed in our first study [23] in healthy persons (mean 72 mcg/L on Rio de Janeiro and 55 mcg/L in Belo Horizonte) and in severe CD patients (65 mcg/L in Rio de Janeiro and 35 mcg/L in Belo Horizonte), as well as the large differences observed in Se levels in diets from diverse Brazilian regions [55], that varied 100 times from one state to the other (e.g., beans in Ceará have 1.2 mcg of Se/g; in São Paulo, 0.016 mcg of Se/g), it is important to explore Se levels in CD patients at different geographical regions, both in Brazil and in other Latin American countries. However, it is difficult to associate low Se serum levels as a cause or a marker (effect) in CCC progression, since the high inflammatory and oxidative stress in severe CCC [35,36,37] would both require and consume selenoproteins [10,18,56]. 

**Figure 3:**
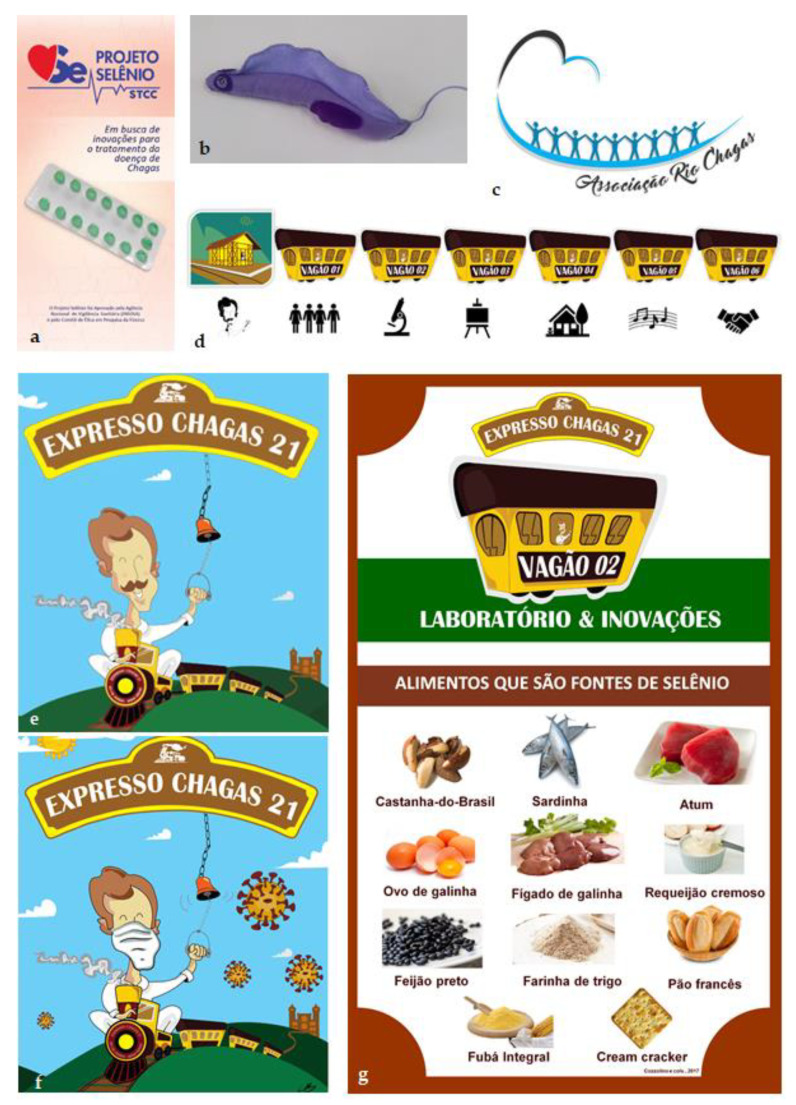
Social legacy from the Selenium Trial for Chagas disease: the project pamphlet (**a**), translation to English: Selenium project STCC, searching for innovations to the treatment of Chagas disease produced in 2015 to invite patients to diverse types of meetings that used a *Trypanosoma cruzi* model (**b**) and education activities that culminated with the organization of a new Chagas disease Association (**c**)—Rio Chagas Association logomark with affected persons (patients, families, and health professionals). (**d**–**g**) show images (**d**–**f**) and banners produced for the “Chagas Express 21” (Expresso Chagas 21), in its expedition version (**d**,**e**,**g**) and in the virtual version (**f**) developed during COVID-19 pandemics (**f**). (**g**) (original words in Portuguese are depicted inside the parentheses) shows an expedition banner indicating “food that are sources of Selenium” (alimentos que são fontes de selênio) as natural diet sources: Brazil nuts (castanha-do-Brasil), fish (sardinha, atum), chicken eggs (ovo de galinha), chicken liver (fígado de galinha), cream cheese (requeijão cremoso), black beans (feijão preto), wheat flour (farinha de trigo), bread, corn meal, cream crackers.

Finally, in addition to our data and the two lessons and implications of STCC, the trial also generated a social legacy (Figure 3): the creation of the Rio Chagas Association and the development of the Chagas Express social technology [57]. These two social spin-offs resulted from STCC after a decision to work on possible secondary social results, related to the translational research assumption that the patients should be central to all the goals in a study [58]. From 2015–2017, many literature data suggested that 100 mcg Se/day was not sufficient to prevent or revert CCC progression, since best results in cardiology parameters were obtained with 200 mcg Se/day [12,51]. Additionally, the necessary changes in the STCC updated protocol [59] reduced the follow-up to one year, instead of the five years initially proposed, and this could limit opportunities to follow more subtle changes in CCC. The choice was then to plan a higher engagement of the participants, using a project pamphlet (Figure 3a) and to propose new meeting options to foster patient´s education about their health conditions and CD issues (Figure 3b). This culminated with the creation of the new Rio Chagas Association (Figure 3c), the first social legacy. The second was the co-creation of a new and original social technology called “Chagas Express 21” (Figure 3d,e), recently presented in a specific paper [57] and in a virtual presentation during COVID-19 pandemics (Figure 3e, available at http://chagas.fiocruz.br/ accessed on 18 February 2022). This technology enables a direct communication with communities and health professionals for education and expansion of diagnostic and therapeutic access in the primary health system.

## 8. Could Selenium Supplementation Improve Chagas Disease Prognosis? Implications for Future Research 

A direct consequence of the low number of basic and clinical studies for innovations in CD treatment (Table 1) is the poor quality of evidence to orient patient health and integral care. The first Brazilian official Clinical Protocol and Therapeutic Guidelines for CD patients, launched in 2018 [46], advised for each recommendation, as well as for the direction of the course of action (performing or not performing the proposed action) and the strength of the recommendation, defined as strong or weak, according to the GRADE system, to be discussed. The first Latin American guidelines for CCC diagnosis and treatment were published by the Brazilian Society of Cardiology in 2011 [60] and 137 recommendations out of 159 (86%) were at level of evidence graded C (data derived from consensual opinions of experts), 21 out of 159 (13%) were graded B level (data derived from less robust meta-analysis, grounded on a single randomized trial or nonrandomized/observational trials), and one recommendation came from multiple significantly sized randomized trials, consistent and/or robust meta-analysis of RCTs. Therefore, consistent with the paucity of robust clinical evidence for all actual CCC treatment guidelines, we propose that most of the data generated in other CVD pathologies under Se supplementation or treatment should be integrated with the few experimental and clinical data already produced for Se treatment in CD. In this perspective, the safety of 100 mcg/day Se administration to CD patients for one year was confirmed in our STCC trial [19] and can be integrated with the safety of 200 mcg/day Se administration to elderly persons in Sweden for four years [12], to the safety of 100 or 200 mcg/day Se administration for five years in the Danish trial for cancer prevention [61], and the safety of 100 mcg/day Se administration for 12 weeks in pregnant women [62]. A recent trial on peripartum cardiomyopathy also confirmed the safety of Se administration [63]. 

A level of evidence graded B could be proposed for Se treatment in CD patients if we consider that the recommendation of Se administration from 100 to 200 mcg/day from 6 months to 5 years to 18 to 88 year-old adults [12,13,19,61,62,63,64,65] is safe. However, a direct beneficial effect of Se treatment in CVD was not definitively proven, and we need to revisit some of the recently sorted reviews (Table S1 in supplementary material) indicating the plausibility of using Se treatments for CVD and CD [14,15,16,17,18,51,52,53]. 

The previously referenced study by Zhang et al. [66] in 2016 analyzed retrospectively 411 patients with heart failure and their serum Se determinations, comparing all-cause mortality and rehospitalization during the follow-up. Patients with the lowest quartile (17.40–44.35 mcg/L Se) were associated with increased risk of all-cause mortality, when compared to those within the higher quartile (94.15–116.7 mcg/L Se), suggesting that “a lower serum Se level was significantly associated with increased risk of all-cause mortality in patients with heart failure”. CCC stages C and D represent those patients (Table 2) and our study in 2002 showed a correlation between Se levels and LFEV decrease [23]. Several selenoproteins are found impaired in suboptimal selenium conditions, potentially aggravating underlying mechanisms such as oxidative stress, inflammation, and thyroid hormone insufficiency [11]. A similar conclusion was found in the meta-analysis conducted by Xiang and co-workers in 2020 [14], which included 12 observational studies or post hoc analyses of RCTs published up to 20 January 2019 and a total of 25,667 individuals: “when analyzed Se level as a continuous variable, each standard deviation Se increase significantly reduced 20% all-cause mortality risk. However, (…) low circulating Se level did not confer significant effect on coronary death”, thus dissociating a general effect of Se levels from specific causes of CVD death. 

The systematic review and meta-analysis of observational studies and RCTs sorted out in 2020 by Kuria et al. [15] expanded the database and found 13 articles out of 1811 that evaluated the association between Se status in the body and CVD incidence and mortality. They arrived at a similar conclusion that “physiologically high Se levels in the body are associated with decreased risk for CVD incidence and mortality. However, people should be cautious about the potential harmful effects from the excessive selenium intake.” Overall, there was a reduced risk of CVD incidence in subjects with physiologically high Se status compared to those with low Se status in the body, and there was a 15% decreased risk of CVD incidence per 10 mcg increment in blood Se concentration. As Al-Mubarak et al. summarized in their review [54], “while the current evidence is not sufficient to advocate selenium supplementation in patients with heart failure, there is a clear need for high level evidence to show whether treatment with selenium has a place in the contemporary treatment of patients with this condition to improve meaningful clinical endpoints.”

The review by Hasani et al. [65] in 2019 studied “all clinical trials which assessed the effect of Se supplementation on antioxidant markers, including oxidative stress index (OSI), antioxidant potency composite (APC) index, plasma malonaldehyde (MDA), total antioxidant capacity (TAC), antioxidant enzymes (superoxide dismutase (SOD), glutathione peroxidase (GPX), catalase (CAT)), and total antioxidant plasma (TAP)”, finding 13 studies among papers searched until June 2017. Results showed that Se supplementation “might reduce oxidative stress by increasing TAC and GPX levels and decreasing serum MDA, both are crucial for reduction of oxidative stress” [65]. This study was followed by the recent review of Zakeri et al. 2021 [66], aiming to evaluate the effects of Se supplementation on oxidative stress biomarkers in adults in 14 studies published from 1995 to 2019. They concluded that “supplementation with Se significantly reduce malondialdehyde levels and increase glutathione and total antioxidant capacity levels”. They emphasized that low dietary intakes of antioxidants and a diet low in fruits and vegetables anticipate increased inflammation and oxidative stress in adolescents and adults and that “current data supports the beneficial effect of Se on hypertension, coronary artery disease, certain cancers and inflammatory diseases.”

Jenkins and co-workers [16] further explored the issue concerning the relevance of Se treatment alone or in combination with other antioxidant mixtures in 2020. The authors identified studies up to 5 June 2020, using the Cochrane Library, Medline, and Embase for potential CVD outcomes, cancer, and all-cause mortality following Se supplementation alone or after antioxidant supplement mixtures with and without Se. They included only RCTs of ≥24 weeks and analyzed data using random-effects models and classified by the Grading of Recommendations, Assessment, Development, and Evaluation approach. A total of 43 out of 9423 studies were used and no association of Se alone or antioxidants was noted with CVD and all-cause mortality. However, a decreased risk with antioxidant mixtures was seen for CVD mortality when Se was part of the mix, with no association when Se was absent. Similarly, a decreased risk was noted for all-cause mortality when Se was part of the antioxidant mixture and of increased risk when Se was absent. The authors suggest that the addition of Se should be considered for supplements containing antioxidant mixtures if they are to be associated with CVD and all-cause mortality risk reduction.

## 9. Conclusions

All the aforementioned recent reviews, meta-analyses and RCTs, supported by experimental studies, strongly suggest that CVD may benefit from Se supplementation and/or treatment, especially in elderly populations [12,13]. The three reviews that discussed the possible involvement of Se deficiency and treatment in CD [67,68,69], complemented by the present work, add arguments that Se supplementation in an acceptable range (34.5 mcg/day) or a little more elevated (100 to 200 mcg/day) could be safely prescribed to CD patients. This agrees with the fact that CD patients are ageing [70] and are vulnerable to the progression of CCC from the main effects of imbalances in oxidative status [21,36,71], as indicated by changes in glutathione peroxidase (GPx), superoxide dismutase (SOD), catalase, glutathione reductase, glutathione S-transferase, reduced glutathione, vitamin E, thiobarbituric acid reactive substances and protein carbonyl [11,71]. Furthermore, there is no risk of toxicity under the threshold of 1 milligram of Se per day [72].

CD has significant economic effects on health systems in Brazil [48] and worldwide, in non-endemic countries that receive immigrants from endemic countries [73], expressed in its complex socio-cultural and economic-political dimensions [74]. CD is a complex multidimensional poverty-related issue. The biomedical and epidemiological dimensions are also complex: genetic diversity of *T. cruzi*, multiple phases and clinical forms of the disease, more than 100 species of vectors, and diversity of mammalian reservoirs impacted by environmental and climate changes. Previously a characteristic disease of the rural population also appears nowadays in urban areas because of the rural exodus [49]. Timely etiological treatment, carried out in Brazil with benznidazole, can delay or prevent complications of chronic CD, but globally, access to diagnosis only occurs in 10% of affected individuals and access to treatment in only 1% [75].

The access to an adjuvant therapy with Se, by formulated supplements or a nutritional approach that could introduce one Brazilian nut per day as a cheap alternative [76] should be kept on the horizon for public health policies proposed to mitigate the CD burden. Treatment with Se has a low cost (less than US$ 10 cents per dose) and the association with coQ10 maintains a positive cost/benefit ratio. Physicians can prescribe Se treatment and follow-up on their CD patients. However, only a randomized, double-blind, phase III, comparative clinical trial can generate robust clinical evidence to support this intervention as public policy. We hope this review can aid inserting these possibilities on the scene and stimulate the creation of an international network to ascertain diverse geographical sites in future studies.

## Figures and Tables

**Table 1:** Number of results found in a general science repository of studies (PubMed) and in a translational research counterpart (Clinical Trial) for Chagas disease, comparing different infections and health areas.

Source ^1^	Key-Word Search	First Year Result	Results Found	% (*)
PubMed	Cancer	1783	4,474,569	100
PubMed	Malaria	1828	102,738	2.2
PubMed	Tuberculosis	1848	271,251	6.0
PubMed	Chagas disease	1945	16,902	0.37
PubMed	HIV	1982	156,469	3.4
PubMed	COVID-19	2019	193,338	4.3
Clinical Trials	Cancer (first year~10,000 studies)	1975	85,383	100
Clinical Trials	Malaria	1982	1215	1.4
Clinical Trials	Chagas disease	1993	75	0.08
Clinical Trials	HIV	1999	8533	9.9
PubMed	Selenium	1909	38,567	100
PubMed	Selenium and cancer	1946	5920	15.3
PubMed	Selenium and heart	1947	1586	4.1
PubMed	Selenium and cardiovascular	1960	1960	5.0
PubMed	Selenium and muscle	1957	1957	5.0
PubMed	Selenium and Chagas disease	1998	25	0.06
PubMed	Translational research	1993	50,124	100
PubMed	Transl. res. and Tropical Medicine	2004	215	0.4
PubMed	Transl. res. and Chagas disease	2010	17	0.03
Clinical Trials	Selenium	1982	350	100
Clinical Trials	Selenium and cancer	1988	65	18.5
Clinical Trials	Selenium and cardiovascular disease	1985	18	5.1
Clinical Trials	Selenium and Chagas disease	2009	1	0.28

* percentages related to a reference number of records found using a major descriptor that is indicated as 100% (cancer, selenium or translational research, according to the section of the Table.; ^1^ Sources: PubMed—https://pubmed.ncbi.nlm.nih.gov/, accessed on 31 October 2021; and Clinical Trials—https://clinicaltrials.gov/, accessed on 31 October 2021).

**Table 2:** Cellular mechanisms underlying pathogenesis of Chronic Chagas disease Cardiopathy and its potential relationship to effects related to selenium levels and/or selenoprotein function and TGF-β signaling.

Mechanism/Evidence—Experimental Studies in Mice	Se	TGF	Ref
Survival rate in the acute phase	Yes	Yes	[24,32,33]
Parasite proliferation change in the acute phase	No	Yes	[24,32]
Myocarditis in the acute phase—enzyme leakage	Yes	Yes	[33,34]
Myocarditis in the acute phase—leukocyte infiltration	Yes	Yes	[27,30,32]
Mycarditis in the chronic phase	Yes	Yes	[25,31]
Fibrosis in the acute phase	nd	Yes	[27,30]
Fibrosis in the chronic phase	nd	Yes	[31]
Oxidative stress at the chronic phase—GPx	Yes	nd	[31,35]
Arrythmia in the chronic phase	Yes	Yes	[25,31]
Heart chambers dilatation in the chronic phase	Yes	nd	[25,31]
**Mechanism/evidence—** **proof of concept in patients**			
Progression of CCC disease in patients	Yes	Yes	[23,26]
Fibrosis in the chronic phase—patients	nd	Yes	[26]
Oxidative stress at the chronic phase –patients GPx	Yes	nd	[36,37]

**Table 3:** Chronic Chagas disease Cardiopathy (CCC) stage according to Brazilian Consensus (2015).

CD Form	ECG	Echo-Cardiogram	Heart Failure	Survival Rate after 5 Years #	Se *	TGF *
NI	Normal	Normal	Absent	100%	72 (*n* = 16)	0.44 (*n* = 12)
IND	Normal	Normal	Absent	100%	70 (*n* = 32)	5.3 (*n* = 22)
**CCC stage**						
A	Altered	Normal	Absent	98%	73 (*n* = 50)	21.4 (*n* = 34)
B1	Altered	Altered, LVEF > 45%	Absent	mild disfunction = 96%		17.8 (*n* = 17)
B2	Altered	Altered, LVEF < 45%	Absent	moderate disfunction = 91%	65 (*n* = 40)	
C	Altered	Altered	Compensable	severe disfunction = 45%	60 (*n* = 11)	
D	Altered	Altered	Refratary	13%		

NI = non-infected; IND = indeterminate form; # Data published in [44]; * Mean values calculated from data obtained in previous studies for Selenium (Se, ng/mL) [23] and Tumor Growth Factor (TGF, ng/mL) [26] in CD patients from Rio de Janeiro. CD = Chagas disease, ECG = electrocardiogram, LVEF = left ventricular ejection fraction, CCC = Chronic Chagas Disease Cardiomyopathy.

## Data Availability

The datasets generated during and/or analyzed during the current study are available from the corresponding author on reasonable request.

## Supplementary Materials

The following supporting information can be downloaded at https://www.mdpi.com/article/10.3390/biom12030349/s1, Table S1: References related to Selenium and myocardiopathies and to Chagas Disease (cited in the references, arranged in chronological order).
