# Supplementary material for: Selenium, TGF-Beta and Infectious Endemic Cardiopathy: Lessons from Benchwork to Clinical Application in Chagas Disease
Source: Biomolecules. 2022 Feb 23;12(3):349. doi: 10.3390/biom12030349 (PMC8944995; doi:10.3390/biom12030349)
Supplement: Supplementary file 1 [file biomolecules-12-00349-s001.zip › biomolecules-1582471-supplementary.pdf]

Table S1: References related to Selenium and myocardiopathies and to Chagas Disease (cited in the references, arranged in chronological order)

Ref	Year	First author /type/ Title	Subject and Se effect<br>LVEF= Left Ventricular Ejection Fraction;<br>CVD= Cardiovascular Disease; HF= Heart Failure;<br>RCT= Randomized Clinical Trial
	**Selenium and cardiopathies**		
53	2005	**Witte** et al (Study RCT)<br><br>*The effect of micronutrient supplementation on quality-of-life and left ventricular function in elderly patients with chronic heart failure*	**Improvement of LVEF in HF**: Analysis of influence of long-term multiple micronutrient (MNT) supplementation (Se 50 mcg) on LVEF, levels of pro-inflammatory cytokines, and quality-of-life (QoL) and 6 min woalking test in elderly patients with CHF (mean 75.4 years). At the end of the follow-up period, LV volumes were reduced in the intervention group with no change in the placebo group. LVEF increased by 5.3+1.4% in the MNT group and was unchanged in the placebo group. Patients taking MNT also had a significant improvement in QoL. Six-minute walk test and inflammatory cytokine levels remained unchanged in both groups. Conclusion was that long-term multiple MNT supplementation can improve LV volumes and LVEF and QoL scores in elderly patients with HF due to LV systolic dysfunction.
11	2014	**Loscalzo** (Review – 43 references)<br><br>*Keshan Disease, Selenium Deficiency, and the Selenoproteome*	**Se and Keshan myocardiopathy:** History of the hypothesis of Se role in Keshan cardiomyopathy (landmarks in 1935, 1962). Efficacy of treatment with sodium selenite in preventing Keshan disease and in mitigating the clinical manifestations in patients with the disease was proven in 1979. In 2003 the selenoproteome was unveiled with 25 selenoproteins. Data implicate a variety of potential mechanisms involving antioxidant selenoproteins that conspire to produce the cardiomyopathy of Keshan disease: "one can readily conclude that Se deficiency promotes oxidant stress and injury, which may also potentiate the oxidant injury of other contributing pathogenic factors, including viral and other infections. The roles of selenium in biologic and pathobiologic processes are intimately related.
66	2016	**Zhang** et al. (Meta-analysis)<br><br>*Selenium status and cardiovascular diseases: meta-analysis of prospective observational studies and randomized controlled trials.*	**Se and CVD** – A meta-analysis to assess the discrepancies between observational and RCT evidence on the effect of Se role in CVD owing to its antioxidant properties. The study included 16 prospective observational studies and 16 RCTs up to 15 December 2013. Results showed a nonlinear relationship of CVD risk with blood Se concentrations across a range of 30-165 [mu]g/l and a significant benefit ofCVD within a narrow selenium range of 55-145 mcg/L. Oral selenium supplements (median dose: 200 [mcg/day]) for 2 weeks to 144 months significantly raised the blood Se concentrations by 56.4 mcg/L, whereas oral selenium supplements (median: 100 mcg/day) for 6 to 114 months caused no effect on CVD. The meta-analysis in prospective studies demonstrated a significant inverse association between Se status and CVD risk within a narrow Se range and a null effect of Se supplementation on CVD was observed in RCTs. These findings indicate the importance of

			considering Se status, dose and safety in health assessment and future study design.
12	2018	**Alehagen** et al<br>(Study: RCT)<br>*Still reduced cardiovascular mortality 12 years after supplementation with selenium and coenzyme Q10 for four years: A validation of previous 10-year follow-up results of a prospective randomized double-blind placebo-controlled trial in elderly*	**Se and CVD** in Sweden (rural municipality) – Se intake is low in Europe, and the endogenous production of coenzyme Q10 decreases as age increases. This study performed an intervention RCT using Se and coenzyme Q10 for 4 years as a dietary supplement. From 443 elderly individuals were included, all CVD mortality (death certificates and autopsy results) After 12 years a significantly reduced CV mortality could be seen in those supplemented with SeCoQ10 (28.1%) x Placebo (38.7%)group. Multivariate Cox regression analysis demonstrated a reduced CV mortality risk in Se group. In those with ischemic heart disease, diabetes, hypertension and impaired functional capacity we demonstrated a significantly reduced CV mortality risk. Study conclusions validate the results obtained in the 10-year evaluation. Since this was a small study, the observations should be regarded as hypothesis-generating.
63	2018	**Rayman** et al.<br>(Study – RCT)<br><br>*Effect of long-term selenium supplementation on mortality: Results from a multiple-dose, randomised controlled trial*	**Se supplement** and CVD- In a Danish population (of relatively low selenium status), the study aimed to determine the effect on mortality of long-term selenium supplementation (5 years) at different dose levels (100, 200, 300 mcg/day). RCT named PRECISE study (single-centre, randomised, double-blinded, placebo-controlled, multi-arm, parallel clinical trial with four groups), enrolling 491 participants aged 60–74 years. Follow-up for 5 years and then mortality for a further 10 years. The study concluded that a 300 mcg/day dose of Se taken for 5 years in a country with moderately-low selenium status and total Se intake over 300 μg/d and high-dose selenium supplements should be avoided.
67	2019	**Hasani** et al.<br>(Review and meta-analysis)<br><br>*Effect of selenium supplementation on antioxidant markers: asystematic review and meta-analysis of randomized controlled trials*	**Se and antioxidant markers –** Aiming to assess the antioxidant effects of Se supplementation. The systematic review and meta-analysis up to June 1, 2017. In total, 13 studies which assessed the effect of Se supplementation on antioxidant markers were included. Therandom-effect meta-analysis method showed that Se supplementation significantly increased GPX and TAC levels and decreased MDA levels. The study concluded that Se supplementation might reduce oxidative stress by increasing TAC and GPX levels and decreasing serum MDA, both of which are crucial factors for reduction of oxidative stress.
14	2020	**Xiang** et al.<br>(Meta-analysis)	**Se and CVD mortality**: meta-anallysis searching for observational studies or post hoc analyses of RCT that evaluated the association between elevated circulating Se level and CVD or all-cause mortality in the general population. 12 observational studies (10 cohort and 2 case-control studies) with a total of

		Circulating Selenium and Cardiovascular or All-Cause Mortality inthe General Population: a Meta-Analysis	25,667 individuals were included. Conclusions were: low circulating selenium level was associatedwith higher risk of cardiovascular or all-causemortality in the general population. Low circulating selenium level did not confer significant effect on coronary death.
15	2020	**Kuria** et al. (Review and meta-analysis)<br><br>*Selenium status in the body and cardiovascular disease: a systematic review and meta-analysis*	**Se and CVD incidence** – Aiming to clarify conflicting evidence on the associations of Se with CVD incidence and mortality, this study analyzed 13 articles (observational studies and RCT), using random-effects model (for effect sizes presented as weighted relative risk (RR) and meta-regression to detect dose-response relationships. Overall, there was a reduced risk of CVD incidence (RR¼0.66; 95% CI:0.40–1.09) and mortality (RR¼0.69; 95% CI: 0.57–0.84) in physiologically high selenium status compared to low selenium status in the body. They found a 15% decreased risk of CVD incidence per 10 mcg increment in blood Se concentration. They concluded that physiologically high Se levels in the body are associated with decreased risk for CVD incidence and mortality. Cautious about the potential harmful effects from excessive intake of Se are stressed.
16	2020	**Jenkins** et al. (Review and meta-analysis)<br><br>*Selenium, ntioxidants, cardiovascular disease, and all-cause mortality: a systematic review and meta-analysis of randomized controlled trials*	**Se and CVD mortality**: This is a systematic review and meta-analysis of 43 RCT to determine the effect of Se supplementation alone and of antioxidant mixtures with or without selenium on the risk of CVD, cancer, and mortality. Results showed that overall, no association of Se alone or antioxidants was seen with CVD and all-cause mortality. However, a decreased risk with antioxidant mixtures was seen for CVD mortality when Se was part of the mix (RR: 0.77; 95% CI: 0.62, 0.97; P = 0.02), with no association when Se was absent. Similarly, when Se was part of the antioxidant mixture, a decreased risk was seen for all-cause mortality (RR: 0.90; 95% CI: 0.82, 0.98; P = 0.02) as opposed to an increased risk when Se was absent (RR: 1.09; 95% CI: 1.04, 1.13; P = 0.0002). Conclusion was that the addition of Se should be considered for supplements containing antioxidant mixtures if they are to be associated with CVD and all-cause mortality risk reduction.
54	2020	**Bomer** et al. (Study - cohort)<br><br>*Selenium and outcome in heart failure*	**Se levels and HF** – Analysis of serum Se levels in BIOSTAT-CHF, a multinational, prospective, observational cohort study that enrolled patients with worsening heart failure, aiming to elucidate if, in clinical practice, moderate selenium deficiency is associated with worse symptoms and outcome in patients with heart failure. Conclusions: Se deficiency in HF patients is independently associated with impaired exercise tolerance and a 50% higher mortality rate, and impaired mitochondrial function in vitro, in human cardiomyocytes. "Clinical trials are needed to investigate the effect of Se supplements in patients with HF, especially if they have low plasma Se levels".
65	2020	**Karaye** et al.	Se in peripartum cardiomyopathy in Nigeria – A study to test the efficacy and safety of Se supplementation in patients who

		(Study – RCT)<br><br>*Selenium supplementation in patients with peripartum cardiomyopathy: a proof-of-concept trial*	had peripartum cardiomyopathy (PPCM) and selenium deficiency. 100 PPCM patients (mean age 28-29 years) with left ventricular ejection fraction (LVEF) < 45% and Se deficiency (< 70 µg/L) were enrolled to receive either oral Se (L-selenomethionine) 200 mcg/day for 3 months or nothing, in addition to the recommended therapy, in an open-label randomised trial, with a follow-up over a median of 19 months. Conclusions were that Se supplementation did not reduce the risk of the primary outcome, but it significantly reduced HF symptoms, and there was a trend towards a reduction of all-cause mortality.
55	2021	**Zhang** et al. (Study-retrospective)<br><br>*The association between serum selenium concentration and prognosis in patients with heart failure in a Chinese population*	Se and HF - Aiming to investigate the association of serum Se level and the outcomes of patients with HF in a Chinese population, this study included 411 patients with HF and serum Se examination retrospectively: 280 survival and 131 deceased patients. Baseline information were collected at patient's first admission and the primary and secondary outcomes were all-cause mortality and rehospitalization for HF during follow-up, respectively. The mean serum level of Se was 68.3 ± 27.7 µg/L. The participants were divided into their serum Se quartiles: 1st quartile (17.4–44.35 µg/L) (n = 104); 2nd quartile (44.35–68.05 µg/L) (n = 102); 3rd quartile (68.05–94.15 µg/L) (n = 103); 4th quartile (94.15–116.7 µg/L) (n = 102). In comparison with the highest quartile, patients with the lowest quartile were associated with increased risk of all cause mortality.
56	2021	**Al-Mubarak** et al. (Review – 103 references)<br><br>*Selenium, Selenoproteins, and Heart Failure: Current Knowledge and Future Perspective*	**Se and HF**. A review with the following subjects: Selenium Deficiency and HF: Current Clinical Knowledge; The definition of "Se deficiency and suboptimal Se status; Observational Studies on Se Status; Se Supplementation Trials; Se and Selenoproteins: Comorbidities in HF Pathology; Transport and Surrogate Marker of Se Status; Oxidative stress and Mitochondrial Dysfunction; Thyroid hormone synthesis and metabolism; Inflammation and Immunity. The summary states that "while the current evidence is not sufficient to advocate selenium supplementation in patients with heart failure, there is a clear need for high level evidence to show whether treatment with selenium has a place in the contemporary treatment of patients with HF to improve meaningful clinical endpoints.
18	2021	**Handy** et al (Review – 146 references)<br><br>*Selenium, a Micronutrient That Modulates Cardiovascular Health via Redox Enzymology*	**Selenoproteins and CV health –** This review foccus on the role of the selenoproteome and protection against oxidative stress. It examines the roles that selenoproteins play in regulating vascular and cardiac function in health and disease, highlighting their antioxidant and redox actions in these processes. Selenoproteins, such as glutathione peroxidase and thioredoxin reductase, play an important role in the reduction of hydrogen and lipid hydroperoxides, and regulate the redox status of Cys in proteins. Emerging evidence suggests a role for endoplasmic reticulum selenoproteins, such as selenoproteins K, S, and T, in mediating redox homeostasis, protein modifications, and endoplasmic reticulum stress. Selenoprotein P, which functions as a carrier of Se to tissues, also participates

			in regulating cellular reactive oxygen species. Cellular reactive oxygen species are essential for regulating cell growth and proliferation, protein folding, and normal mitochondrial function, but their excess causes cell damage and mitochondrial dysfunction, and promotes inflammatory responses. Authors review experimental evidence for a role of individual selenoproteins in CVD, by modulating the damaging effects of reactive oxygen species.
13	2021	**Giacconi** et al. (Study – cross-sectional) *Reduced levels of plasma selenium are associated with increased inflammation and cardiovascular disease in an Italian elderly population*	Low Se associated to CVD in Italy – Aiming to investigate the association between Se status, CVD risk, cardio-metabolic and inflammatory markers in elderly population (858 control subjects -mean age 73.4 and 606 CVD patients -mean age 72.5. Se< 60 mcg/L was considered as Se deficiency. A multivariate logistic regression showed that Se deficiency was associated with an increased CVD risk (odds ratio=1.946, 95% CI: 1.19–3.18, p < 0.01), leading to the conclusion that Se deficiency is independently associated with CVD, and with elevated circulating inflammatory markers and affects the expression of cytokines, chemokines and sirtuins in PBMCs.
68	2021	**Zakeri** et al. (Review - RCT) *Selenium Supplementation and Oxidative Stress: A review*	Se and oxidative stress: an imbalance between the production of oxidants (free radicals or reactive oxygen species) and their eradication by protective mechanisms, such as antioxidants (vitamin C, vitamin E, b-carotene, selenium and methionine). This review detected 14 studies that evaluated the effect of selenium supplementation on oxidative stress biomarkers n adults. The conclusions were that several studies have shown that supplementation with selenium significantly reduce Malondialdehyde levels and increase Glutathione and total antioxidant capacity levels.
17	2022	**Yang** et al. (Review and meta-analysis) *Selenium concentration is associated with occurrence and diagnosis of three cardiovascular diseases: A systematic review and meta-analysis*	Se and HF /other CVD - This meta-analysis aimed to compare and analyze differences in Se levels between patients with heart failure (HF), myocardial infarction (MI), coronary heart disease (CHD), and healthy people. A total of 49 studies (including 61 cohorts) were included, up to November 20, 2020. Results showed that there was a significant difference in Se levels between HF, MI, CHD patients and healthy people. The standard mean difference (SMD) level of Se in HF patients, MI patients, and CHD patients were all significantly lower compared to healthy controls. Se levels in HF and controls was positively correlated with time. Se level was found to be a good diagnostic marker of MI. The conclusion was that Se levels in patients with HF, MI, and CHD are generally lower compared with healthy controls. However, due to the small number of included studies, further studies are needed to confirm the present results.
10	2022	**Tsuji** et al. (Review – 157 references)	This review highlights tha role os Se as an essential element in the diet of humans, all other mammals, and many other life forms, its health benefits (preventing CVD and certain forms of cancer, slowing AIDS progression in HIV patients, supporting

		Historical Roles of Selenium and Selenoproteins in Health and Development: The Good, the Bad and the Ugly	male reproduction, inhibiting viral expression, and boosting the immune system). The review features (1) how this element makes its way into protein as the 21st amino acid in the genetic code, selenocysteine (Sec); (2) the vast amount of machinery dedicated to synthesizing Sec uniquely on its tRNA; (3) the incorporation of Sec into protein; and (4) the roles of the resulting Sec-containing proteins (selenoproteins) in health and development.
58	2022	**Bomer** et al. (Review – 191 references) *Micronutrient deficiencies in heart failure: Mitochondrial dysfunction as a common pathophysiological mechanism?*	This review foccus on micronutrients (MNT), including coenzyme Q10, zinc, copper, selenium and iron, that are required to efficiently convert macronutrients to ATP, the main cardiac fuel. It discusses that increasing the delivery of energy substrates (e.g., fatty acids, glucose, ketones) to the mitochondria will be worthless if the mitochondria are unable to turn these energy substrates into energy. It states that up to 50% of patients with hHF are deficient in one or more micronutrients in cross-sectional studies and that new strategies to treat or prevent HF are urgently needed. Authors defend that MNT deficiency has a high impact on mitochondrial energy production and should be considered an additional factor in the heart failure equation, moving our view of the failing myocardium away from an "an engine out of fuel" to "a defective engine on a path to self-destruction." They suggest that supplementation with MNT, preferably as a package rather than singly—might be a potential therapeutic strategy in the treatment of HF patients.
**Selenium and human Chagas disease**			
25	2002	Rivera et al.	Lower Se levels in severe CCC
28	2002	Araujo-Jorge et al.	Higher TGF-b levels in severe CCC
39	2003	Pérez-Fuentes et al.	High inflammatory and oxidative stress in CCC
38	2006	Wen et al.	Mitochondrial disfunction in CCC
52	2014	Brasil et al	STCC RCT protocol for CCC
61	2018	Holanda et al.	Updated STCC RCT protocol for CCC
19	2021	Holanda et al.	STCC results in CCC after one year treatment and follow-up
**Selenium and experimental Chagas disease (mice)**			
34	1998	Davis et al.	Beneficial effect of selenium supplementation in acute phase
26	2002	Souza et al.	Se deficiency increases mortality in acute phase
27	2003	Souza et al	Se treatment prevents cardiac injuries in acute phase
37	2004	Malvezi et al.	High inflammatory and oxidative stress
27	2010	Souza et al.	Se treatment prevents cardiac injuries in chronic phase
